# Eccentric Exercise and the Critically Ill Patient

**DOI:** 10.3389/fphys.2017.00120

**Published:** 2017-02-28

**Authors:** W. Kyle Mitchell, Tanja Taivassalo, Marco V. Narici, Martino V. Franchi

**Affiliations:** ^1^MRC-ARUK Centre of Excellence for Musculoskeletal Ageing Research, School of Medicine, University of NottinghamDerby, UK; ^2^Department of Kinesiology and Physical Education, McGill UniversityMontreal, QC, Canada; ^3^Department of Physiology and Functional Genomics, Myology Institute, University of FloridaGainesville, FL, USA

**Keywords:** eccentric exercise, critically ill patients, eccentric contraction, intensive care, intensive care units

Critically ill patients demonstrate established or impending multi-organ failure which may be due an acute condition such as sepsis or trauma, a deterioration in a chronic condition, or failure to progress during the recovery process and is often in part due to intrinsic risk factors such as age and concomitant disease processes (Loftus and Royal College of Surgeons of England, 2010). Survivors of critical illnesses commonly experience persisting, and sometimes permanent, disability because of loss of muscle mass, compromised muscle function, and the subsequent loss of strength (Iwashyna et al., 2010; Herridge et al., 2011). This process is detectable from early in the course of the illness. Human diaphragm shows decreased fiber cross-sectional area and altered gene expression with <2 days of mechanical ventilation (Levine et al., 2008). Healthy volunteer studies show that within 5 days of leg immobilization there is a detectable decrease in quadriceps cross-sectional area and strength (Dirks et al., 2014), muscle weakness is observed in patients suffering sepsis, acute pancreatitis (Gordon et al., 2013), trauma, burns (Cioffi, 2001), and the post-operative period, especially in those who have experienced complications of surgery. Weakness and loss of muscle mass significantly predict mortality in older people and patients with conditions such as chronic obstructive pulmonary disease (COPD; Marquis et al., 2002; Landi et al., 2005). A potentially lethal combination of immobilization and inflammation combine to achieve rapid and often catastrophic atrophy (Rudrappa et al., 2016). Sarcopenia and cachexia of chronic illness put the older patient with significant co-morbidities at greatest risk (Unroe et al., 2010). Poor mobility and weakness are established predictors of poor outcomes in critically ill patients (Kasotakis et al., 2012).

Future advances in the management of the critically ill patient demand effective therapies to minimize the decrement in skeletal muscle function. Evidence exists to support a role for early physical therapy or rehabilitation in minimizing weakness and muscle atrophy of critical illness (Adler and Malone, 2012; Hashem et al., 2016). However, compliance with exercise programs may be reduced by common features of critical illness such as the circulatory instability associated with decreased systemic resistance and vasopressor administration or compromised gas exchange with acute respiratory distress syndrome and hospital-acquired/ventilator-associated pneumonia. Strategies to maximize compliance with, and benefit from, physical therapy in the critically ill patient should take these complicating features into account and provide maximal anabolic stimulation whilst not exceeding the compromised cardiorespiratory capacity of the patient. The authors propose that exercise paradigms that rely predominantly *eccentric actions* may circumvent these barriers to strength training.

The metabolic demand of generating force during muscle lengthening, i.e., eccentric activity (ECC) is greatly reduced compared to generation of similar force during muscle shortening, i.e., concentric activity (CON; Fenn, 1924; Abbott et al., 1952; Bassett, 2002). ECC actions classically form part of braking or actions to lower a load with gravity, such as stair descent or downhill walking. For the same velocity of contraction (considered *negative* for ECC and *positive* for CON), muscle force generation in ECC contractions exceeds that of CON contraction: observations in healthy humans showed that ECC contractions are associated to greater forces than CON ones, as ECC resulted *in vivo* 1.2–1.4 times greater than isometric and CON peak values (Westing et al., 1991; Franchi et al., 2014), and up to 1.6 times greater when comparing ECC vs. CON isokinetic strength (Hollander et al., 2007).

Exercise elicits a cardiovascular response, increasing both heart rate and stroke volume, and thus cardiac output (Dufour et al., 2004). Particularly for aerobic exercise, it has been shown that these cardiovascular responses are largely driven by muscle metabolic demands; specifically the increased rate of oxygen uptake (VO_2_) that reflects the increase in oxygen utilization within the mitochondrial respiratory chain that is the eventual consequence of the activation of myosin ATPase activity of type I and type IIA muscle fibers (Dufour et al., 2007). A smaller element of the cardiovascular response to muscle activity appears to be dependent directly upon the generation of muscle mechanical tension (Kaufman and Hayes, 2002; Dufour et al., 2007). The increment in VO_2_ with CON activity is four- to five-fold greater than that seen with mechanical-power matched ECC activity (Abbott et al., 1952; Dufour et al., 2004). It is therefore possible to dissociate power/ force generation from metabolic demand (VO_2_) and subsequent cardiovascular responses. This has previously been reviewed (Isner-Horobeti et al., 2013).

Intriguingly, the reduced metabolic impact of ECC compared to CON is not matched by a reduced tendency to achieve structural or functional muscle adaptation to training. Mechanical loading of contracting muscle stimulates maintenance, or even gain, of muscle mass via signaling pathways including the mammalian target of rapamycin complex 1 (mTORC1); these in turn promote muscle protein synthesis (Hornberger and Chien, 2006). ECC training achieves greater acute activation of mitogen-activated protein kinase (MAPK) pathways (both stress-activated protein kinase, p38, extracellular signal-regulated kinase 1 and 2, ERK1/2 and p90RSK) than does CON, in animals (Wretman et al., 2001) and humans (Franchi et al., 2014). Because ECC exercise may achieve generation of greater tension forces within muscle (Narici et al., 1989; LaStayo et al., 2003), and because MAPKs and mechanosensitive protein focal adhesion kinase (FAK) activation has been found to be quantitatively related to tension (Martineau and Gardiner, 2002; Li et al., 2013), this specific loading typology could potentially result in greater efficiency in maintaining muscle mass, at much lower exercise metabolic cost.

Training with ECC contractions, when performed at high intensities, is commonly associated with greater increases in total and eccentric strength compared to CON resistance training (Roig et al., 2009; Isner-Horobeti et al., 2013). The mechanisms regulating changes in muscle size in response to ECC vs. CON resistance exercise have not yet been fully elucidated, especially in humans. There is still some debate regarding the singular contribution of both contraction types to muscular hypertrophy (Douglas et al., 2016). As lengthening contractions have the possibility to generate greater muscle force than isometric and shortening ones (Westing et al., 1991), the common belief is that eccentric exercise may promote larger increases in muscle size compared to concentric and isometric training (Roig et al., 2009). However, other authors (Wernbom et al., 2007; Hyldahl and Hubal, 2014) have suggested that if the two types of loading are performed at same intensity and/or work volume, then it is difficult to establish which is the best training mode, as significant hypertrophy is reached in either case. Indeed, similar increases in muscle mass have been previously reported between ECC and CON resistance training (Franchi et al., 2014, 2015; Hyldahl and Hubal, 2014).

Nonetheless, in spite of the similar hypertrophy, the *pattern* of muscle growth with ECC loading has been shown to be substantially different from that of CON loading (Franchi et al., 2014; Narici et al., 2016). Investigations into the architectural adaptations of human skeletal muscle to ECC and CON training have shown that ECC loading promotes muscle growth through addition of sarcomeres in series, while CON training seems to mainly promote addition of sarcomeres in parallel (Reeves et al., 2009; Franchi et al., 2014, 2015). Differential muscle growth in response to CON and ECC loading reflects differential activation of molecular signaling pathways regulating muscle growth (as explained above). Moreover, the differential pattern of growth may have a functional correlate, as a longer fascicle length (and thus more sarcomeres placed in-series) is correlated to greater muscle contraction velocities, whereas more sarcomeres placed in-parallel (likely resulting in larger physiological cross-sectional area), will be proportional to greater muscle force (Lieber and Fridén, 2001).

Unaccustomed, high intensity ECC exercise can cause myofibrillar damage, local, and systemic inflammatory responses, delayed onset muscle soreness, initial loss of strength (Fridén et al., 1983) and even insulin resistance (Kirwan et al., 1992). This has perhaps discouraged consideration of the potential benefit of ECC exercise in populations considered to be at high risk such as the frail, elderly, and medically co-morbid. However, it should be stressed that detectable muscle damage and inflammation are neither inevitable nor prerequisites for the beneficial structural and functional changes seen with ECC training. Adaptation can be achieved without apparent damage or inflammation (Flann et al., 2011). Gradually progressive training intensity can avoid muscle damage and associated soreness in healthy and disease populations (LaStayo et al., 2003, 2014; Rocha Vieira et al., 2011).

There has been some recognition that exercise modalities that make use of an ECC component may have benefit in clinical populations. A wide range of techniques and equipment such as upper and lower limb ergometers exist to facilitate exercise that achieves predominantly ECC contractions (Isner-Horobeti et al., 2013; Hoppeler, 2016). Their application to date has focused on rehabilitation of specific musculoskeletal conditions and, to a lesser extent, on some chronic conditions. These include COPD, heart failure (HF), coronary artery disease, type 2 diabetes, survivors of breast, prostate and colon cancer, and neurological conditions including Parkinson's disease, multiple sclerosis and stroke (LaStayo et al., 2014). Patients with severe COPD have been shown to tolerate progressively increasing intensity ECC cycling with excellent compliance and no side effects (Rocha Vieira et al., 2011). In HF, ECC training at low rates of perceived exertion is well-tolerated and in a prospective randomized control trial, a 30 min ECC bout 3 times per week for 7 weeks can achieve an increase in muscle strength, not seen in controls undertaking perceived-intensity matched CON training (Casillas et al., 2016).

However, the authors are unable to find any reports employing ECC exercise training in patients with acute systemic illnesses, or critical care needs, despite the well-established, disappointing long-term outcomes associated with muscle weakness in these patients. Numerous factors may contribute to the lack to studies into ECC exercise training in critically ill patients. The assumption that bedrest is a key element in the recovery period has only been effectively challenged within the last few decades, with the demonstration of the effectiveness of aggressive rehabilitation and early ambulation as components of “enhanced recovery” approach to major surgery (Varadhan et al., 2010). Although examples exist of equipment that facilitates ECC training (Isner-Horobeti et al., 2013), these are often bespoke and financial constraints may limit the feasibility of providing these facilities to clinic populations. Increasing rigor in clinical governance and research ethics approval pathways and risk aversion within health care institutions may also impede efforts to undertake novel exercise regimens in patients. Finally, the authors perceive a low awareness amongst clinicians of the potential for ECC training to circumvent barriers to strength training in the critically ill patient. Skepticism or ignorance about a novel physical therapy on the part of a responsible clinician may hamper efforts to recruit for such studies.

Training paradigms that minimize cardiovascular and respiratory demands whilst promoting maintenance, or decelerating the expected rapid loss, of muscle mass and strength are ideally suited for use by the critically ill patient, and for introduction at the earliest stages of rehabilitation. Whilst ECC exercise has been explored in the treatment of several specific injuries and chronic conditions, the potential for systemic benefit to the critically ill patient has been overlooked. Specific questions that remain unanswered include the extent to which the benefits of ECC exercise can be reproduced in the presence of the vigorous systemic inflammatory response that usually accompanies critical illness and which is known to compromise the responsiveness of patients to existing exercise therapies (Walsh et al., 2015; Griffith et al., 2016; Norheim et al., 2017).

The theoretical benefits of exercise regimens designed around eccentric contractions to the critically ill patient demand that this be considered in future studies of physical therapy and rehabilitation.

## Author contributions

Conception of the manuscript: WM, MF, MN. All the authors equally contributed to the writing process of the present manuscript.

### Conflict of interest statement

The authors declare that the research was conducted in the absence of any commercial or financial relationships that could be construed as a potential conflict of interest.
